# Clinical Outcomes After Use of Inhaled Corticosteroids or Oral Steroids in a COVID-19 Telemedicine Clinic Cohort: Retrospective Chart Review

**DOI:** 10.2196/36023

**Published:** 2023-02-23

**Authors:** Michele Cellai, Jodi Roberts, Miranda A Moore, Nikhila Gandrakota

**Affiliations:** 1 General Internal Medicine Seavey Clinic School of Nursing Emory University Atlanta, GA United States; 2 General Internal Medicine Emory Healthcare Atlanta, GA United States; 3 Department of Family and Preventive Medicine School of Medicine Emory University Atlanta, GA United States; 4 Family Medicine Residency Program School of Medicine Emory University Atlanta, GA United States; 5 Department of Medicine School of Medicine Emory University Atlanta, GA United States

**Keywords:** COVID-19, lung, post-acute sequela, steroid use, ICS, pandemic, therapy, treatment, steroid treatment, COVID-19 treatment, patient outcome, pulmonary, COVID symptoms, telehealth

## Abstract

**Background:**

COVID-19 concerns remain among health care providers, as there are few outpatient treatment options. In the early days of the pandemic, treatment options for nonhospitalized patients were limited, and symptomatic treatment and home-grown guidelines that used recommendations from the Global Initiative for Asthma Management and Treatment were used.

**Objective:**

The possibility that inhaled corticosteroids (ICS) might reduce the risk of respiratory symptoms and promote recovery was the impetus for this review, as it has already been shown that in the nonhospitalized patient population, oral corticosteroids (OCS) in the acute phase could have an adverse effect on recovery. We investigated if (1) patients treated with ICS were less likely to require referral to a post–COVID-19 clinic or pulmonary specialist than patients without ICS treatment or with OCS therapy, and (2) if OCS use was associated with worse health outcomes.

**Methods:**

In a retrospective chart review, we identified all patients with acute illness due to COVID-19 that were followed and managed by a telemedicine clinic team between June and December 2020. The data were electronically pulled from electronic medical records through April 2021 and reviewed to determine which patients eventually required referral to a post–COVID-19 clinic or pulmonary specialist due to persistent respiratory symptoms of COVID-19. The data were then analyzed to compare outcomes between patients prescribed OCS and those prescribed ICS. We specifically looked at patients treated acutely with ICS or OCS that then required referral to a pulmonary specialist or post–COVID-19 clinic. We excluded any patients with a history of chronic OCS or ICS use for any reason.

**Results:**

Prescribing ICS during the acute phase did not reduce the possibility of developing persistent symptoms. There was no difference in the referral rate to a pulmonary specialist or post–COVID-19 clinic between patients treated with OCS versus ICS. However, our data may not be generalizable to other populations, as it represents a patient population enrolled in a telemedicine program at a single center.

**Conclusions:**

We found that ICS, as compared to OCS, did not reduce the risk of developing persistent respiratory symptoms. This finding adds to the body of knowledge that ICS and OCS medications remain potent treatments in patients with acute and postacute COVID-19 seen in an outpatient setting.

## Introduction

The global pandemic caused by SARS-CoV-2 and resultant COVID-19 has caused significant morbidity and mortality all around the world. Millions have recovered from COVID-19 worldwide despite limited treatment options in both the inpatient and outpatient settings. Concern remains among health care providers that although 80% of patients that contract COVID-19 are not hospitalized, there are few outpatient treatment options. Additionally, approximately 30% of patients have been identified as having at least one persistent symptom as a result of having contracted COVID-19 [1]. Fatigue and dyspnea are the most common persistent symptoms in older adults [2], and memory issues are also common. At this time, we do not have an effective treatment to prevent persistent symptoms of COVID-19. This study was undertaken in the hope of identifying a treatment regimen that might reduce the burden of persistent respiratory symptoms after recovering from acute COVID-19.

While treatments for COVID-19 in hospitalized patients have been developed from repurposed medications, such as remdesivir [3], treatments on the outpatient side have been difficult to identify. Treatment has focused on mitigation of symptoms. Research into the use of dexamethasone in hospitalized patients with moderate to severe COVID-19 does show benefit; however, dexamethasone is not recommended for use in the outpatient setting in mild to moderate cases of COVID-19 that do not require oxygen support, as research has shown potential harm [4]. To date, the only approved treatments for COVID-19 in the outpatient setting have included, early on, monoclonal antibody therapy and later, antiviral therapy. This highlights the challenge of treating patients in the outpatient realm.

The inflammatory response to SARS-CoV-2 appears to contribute significantly to morbidity and mortality [5]. Corticosteroids are potent anti-inflammatory medications that suppress inflammation but can also suppress the immune system [6]. ICS reduce inflammation, swelling, and mucous production in the lungs. Further investigation into how the use of corticosteroids might modify the outcome of SARS-CoV-2 infection and the development of prolonged COVID-19 symptoms is needed. Recent research into the use of ICS after COVID-19 diagnosis showed that the early administration of inhaled budesonide improved outcomes by reducing the likelihood of needing urgent medical care and reducing the time to recovery after early COVID-19 [7]. Additionally, there was a relative reduction of 91% in clinical deterioration among study patients receiving ICS. Therefore, evaluation of the use of ICS in the treatment of outpatient COVID-19 and long-term outcomes is advised. We hypothesized that patients treated with ICS would be less likely to require referral to a post–COVID-19 clinic or pulmonary specialist than patients without ICS treatment or patients that received OCS therapy, and we also hypothesized that OCS use would be associated with worse health outcomes.

## Methods

### Study Design

We conducted a retrospective chart review of nonhospitalized adult patients newly enrolled at the Emory Virtual Outpatient Management Clinic (VOMC) in Atlanta, Georgia, for COVID-19 (confirmed by nasopharyngeal sampling) between June 1, 2020, and December 31, 2020. The Emory Healthcare post–COVID-19 clinic is a multidisciplinary team of providers from the pulmonary, cardiology, and neurology divisions. Patients may be referred for any persistent symptoms associated with COVID-19 illness. The study inclusion criteria included (1) enrollment in the telemedicine clinic within 28 days of a positive COVID-19 test (confirmed with a nasopharyngeal polymerase chain reaction test) and (2) a specific symptom onset date that was recorded at the time of enrollment. The exclusion criteria included (1) hospitalization prior to telemedicine clinic enrollment and (2) hospitalization after enrollment, if the patient was treated with antiviral medication and steroids. Patients with chronic steroid use (n=20) were also excluded from the post–COVID-19 clinic referral evaluation, as the goal was to review acute steroid use outcomes.

The chart review included (1) verification of patient demographics, (2) verification of symptom onset date (from the screening clinic notes and the telemedicine clinic enrollment notes), and (3) review of follow-up notes with the pulmonary division or post–COVID-19 clinic. Charts were also reviewed through April 30, 2021, to evaluate them for future referrals to the pulmonary division or the post–COVID-19 clinic. Descriptive statistics were calculated using Stata/SE (version 16; StataCorp).

### Ethical Considerations

This work was deemed to be a clinical quality improvement project and not human subject research by the Emory University Institutional Review Board. Data were extracted from electronic health records and patient names were removed prior to analysis.

## Results

During the specified period, a total of 1167 unique visits from patients newly enrolled at the Emory VOMC who met the inclusion criteria were identified, including 805 women (67%) and 362 men (Table 1). The average age was 48.54 (range 18-96) years. Overall, 9.6% of the patients had preexisting asthma (n=112), which is slightly more than the US average for adults of 8.4% [8]; 0.9% had COPD (n=10), which is less than the US average of 5.6% [9]; and 3.1% had other lung diseases (n=36). Unfortunately, information on preexisting conditions was missing for approximately one-third of the cohort, so these averages may be skewed.

**Table 1:** Characteristics of COVID-19 patients (N=1167) seen at the Emory Healthcare Virtual Outpatient Management Clinic from June 1 through December 31, 2020.

Demographics			Values
**Age (years)**			
	Mean (SD)	48.54 (15.62)	
	Median (range)	49 (18-96)	
**Age categories (years), n (%)**			
	18-29	152 (12.97)	
	30-39	215 (18.34)	
	40-49	249 (21.25)	
	50-59	243 (20.73)	
	60-69	192 (16.38)	
	70-79	87 (7.42)	
	≥80	34 (2.9)	
**Sex, n (%)**			
	Female	805 (68.98)	
	Male	362 (31.02)	
**Past medical history, n (%)**			
	Asthma	112 (19.68)	
	Chronic obstructive pulmonary disease	10 (1.87)	
	Lung disease	36 (6.68)	
**Acute steroid prescription history, n (%)**			
	ICS^a^	33 (2.8)	
	OCS^b^	34 (2.9)	
	ICS and OCS	14 (1.1)	
**Chronic steroid prescription history, n (%)**			
	ICS	8 (0.7)	
	OCS	8 (0.7)	
	ICS and OCS	4 (0.3)	
	None	1066 (91.3)	

^a^ICS: inhaled corticosteroids.

^b^OCS: oral corticosteroids.

Of 1167 patients, 33 of 1167 (2.8%) were newly prescribed ICS and 34 of 1167 (2.9%) were prescribed OCS; 14 (1.2%) were prescribed both ICS and OCS (Table 1). For patients requiring referral to the post–COVID-19 clinic or to a pulmonary specialist due to persistent COVID-19 respiratory symptoms as of April 30, 2021, 3 of 33 (9.1%) were from the group receiving ICS, 4 of 34 (11.8%) were from the group receiving OCS, and 4 of 14 (28.6%) from the group that received both OCS and ICS. Additionally, 1 of 20 (5%) with chronic corticosteroid use, and 13 of 1066 (1.2%) without corticosteroid use (Table 2).

**Table 2:** Acute steroid use and post–COVID-19 clinic follow up among patients (N=1167) seen at the Emory Healthcare Virtual Outpatient Management Clinic from June 1 through December 31, 2020.

Steroid use			Patients visiting the post–COVID-19 clinic, n (%)
**Acute**			
	Inhaled corticosteroids (n=33)	3 (9.1)	
	Corticosteroids (n=34)	4 (11.8)	
	Oral and inhaled corticosteroids (n=14)	4 (28.6)	
Chronic corticosteroids (n=20)			1 (5)
None (n=1066)			13 (1.2)

## Discussion

### Principal Findings

Our data demonstrates that an equal number of patients prescribed ICS versus OCS were eventually referred to the post–COVID-19 clinic or a pulmonary specialist. Additionally, there was a small group of patients that received both ICS and OCS with a higher percentage requiring referral. As our hypothesis was that ICS would reduce the risk of persistent post–COVID-19 symptoms compared to OCS, this finding was unexpected.

### Comparison With Prior Work

A total of 11 of 1167 (0.94%) patients required referral for persistent symptoms due to COVID-19, notably fewer than in previously published data, which indicate that 8% to 30% of people that contract COVID-19 and are not hospitalized continue to have symptoms 6 months after initial infection [10,11]. The recent publication of research showing that inhaled budesonide improved outcomes by reducing the likelihood of needing urgent medical care, as well as by reducing time to recovery after early COVID-19, is an important consideration for early care but does not address the development of persistent respiratory symptoms. Of note, 1 of the 20 patients with chronic steroid use required referral to a post–COVID-19 clinic as well as 13 of 1066 (1.2%) patients not treated with any steroids.

### Future Directions

Future studies into the treatment of COVID-19 symptom mitigation and the reduction of long-term sequelae should include evaluation of standardized dosing regimens for oral and inhaled corticosteroids, such as a standard 6-day oral steroid taper of methylprednisolone and several weeks to months of inhaled fluticasone at 2 puffs 2 to 3 times a day. Additionally, comparing early ICS treatment with OCS use after 10 to 14 days could also be an area of future study.

### Limitations

These data represent a specific patient population enrolled in a telemedicine program at a single center and may not be generalizable to other populations. As mentioned above, past medical history data were missing for approximately 30% of the cohort due to changes in data collection methods during the period reviewed. Thus, the population studied may differ in chronic condition status from the general population. Additionally, the ICS and OCS regimens were not standardized. Some patients receiving ICS received nebulized budesonide, while others received fluticasone or budesonide via hydrofluoroalkane inhaler or fluticasone via dry powder disk. Although pre–COVID-19 ICS and OCS regimens were not standardized for acute or subacute viral illness, variability in medications and dosing could have affected the outcomes.

### Conclusion

Our review did not reveal a difference in the number of patients developing persistent respiratory symptoms and requiring referral to a post–COVID-19 clinic or pulmonary specialist after acute COVID-19 when OCS was prescribed, as compared to ICS. Thus, the prescribing of ICS or OCS should not be avoided. Although previous research has shown that OCS may be harmful, our review did not reveal a difference in the need for referral for persistent symptoms for patients who were prescribed OCS. The previous research on ICS, specifically on inhaled budesonide, clearly shows a short-term benefit but does not address long-term outcomes. Our research shows that ICS, as compared to OCS, did not reduce the risk of developing persistent respiratory symptoms. Although there are myriad reasons to choose one delivery route over another, including cost, side effects, ease of use, and patient preference, our results add to the body of knowledge that ICS and OCS medications remain a potent treatment for outpatients with acute and postacute COVID-19.

### Treatment Considerations

As ICS show clear benefit early in the course of acute COVID-19, they should be prescribed for respiratory symptoms. Cost may be a factor, and budesonide inhaled 3 times per day via nebulizer may be a more affordable option than a hydrofluoroalkane inhaler or dry powder inhaler used 2 or 3 times a day. Based on previous research, OCS may be considered for persistent (beyond 14 days) respiratory symptoms if cost is an issue and ICS are financially not an option.

## Abbreviations

ICS: inhaled corticosteroids
OCS: oral corticosteroids
VOMC: Virtual Outpatient Management Clinic

## Data Availability

Data sets generated during and/or analyzed during the current study are available from the corresponding author on reasonable request.
